# Study of Factors Involved in Tongue Color Diagnosis by Kampo Medical Practitioners Using the Farnsworth-Munsell 100 Hue Test and Tongue Color Images

**DOI:** 10.1155/2014/783102

**Published:** 2014-04-06

**Authors:** Takeshi Oji, Takao Namiki, Toshiya Nakaguchi, Keigo Ueda, Kanako Takeda, Michimi Nakamura, Hideki Okamoto, Yoshiro Hirasaki

**Affiliations:** ^1^Department of Japanese Oriental (Kampo) Medicine, Graduate School of Medicine, Chiba University, 1-8-1 Inohana, Chuo-ku, Chiba 260-8670, Japan; ^2^Center for Frontier Medical Engineering, Chiba University, 1-33 Yayoi-cho, Inage-ku, Chiba 263-8522, Japan; ^3^Graduate School of Engineering, Chiba University, 1-33 Yayoi-cho, Inage-ku, Chiba 263-8522, Japan

## Abstract

In traditional Japanese medicine (Kampo medicine), tongue color is important in discerning a patient's constitution and medical conditions. However, tongue color diagnosis is susceptible to the subjective factors of the observer. To investigate factors involved in tongue color diagnosis, both color discrimination and tongue color diagnosis were researched in 68 Kampo medical practitioners. Color discrimination was studied by the Farnsworth-Munsell 100 Hue test, and tongue color diagnosis was studied by 84 tongue images. We found that overall color discrimination worsened with aging. However, the color discrimination related to tongue color regions was maintained in subjects with 10 or more years of Kampo experience. On the other hand, tongue color diagnosis significantly differed between subjects with <10 years of experience and ≥10 years of experience. Practitioners with ≥10 years of experience could maintain a consistent diagnosis of tongue color regardless of their age.

## 1. Introduction

In traditional Japanese medicine (Kampo medicine), observing the tongue shapes and colors is a method for diagnosing the patient's constitution and medical conditions. In addition to the information that the tongue reveals, Kampo evaluations are supplemented with data from questionnaires, pulse, and abdominal diagnosis. Tongue diagnosis is particularly useful for detecting* Mibyou*, the “disease-oriented” healthy stage in Kampo medicine. Kampo tongue information, such as tongue pain in the dental oral area, can be used to prevent potentially refractory diseases [1, 2]. Generally, tongue diagnosis focuses on tongue texture and tongue coating. The colors and shapes of each part can be investigated to diagnose medical conditions.

Mainly, tongue color is the result of light reflection and light absorption. The color of the tongue (tongue color) is especially dependent on internally diffused light. Tongue color diagnosis (TCD) can provide very useful information for medical conditions. By TCD, we can get useful information about the patient's reservoirs of heat and cold, exhaustion level, mental state, digestive system function, blood circulation dynamics, and water metabolic state. However, tongue color diagnosis is affected by two types of factors. One is environmental factors, such as light sources or room temperature, which influence impact diagnosis. The other type includes the subjective factors of the observers, especially their knowledge of Kampo and experience using it.

In recent years, to solve the problem of environmental factors (EF), many researchers have developed a tongue imaging system that operates at constant conditions [3–6]. Chiu devised the hardware and software for tongue imaging and examined the tongue surface and tongue coating, divided into areas related to concepts of traditional medicine [3]. Wang et al. introduced a method of evaluating the color of the tongue surface, dividing the tongue into different regions and excluding the tongue coating [4, 5]. Zhang et al. devised a system for tongue imaging and quantifying the tongue image information, considering both measurement values and the patient's past medical history [6]. Kanawong et al. compared the color value of tongue images with the patient's hot or cold condition [7]. In addition, they measured the RBG value of tongue images obtained in the same imaging environment and evaluated the tongue color quantitatively then used those data for early detection of diseases such as appendicitis and liver cancer [8, 9]. We first undertook research to standardize tongue diagnosis in 2008. As one of our research outcomes, we have been developing a new tongue imaging method and diagnostic support system (Tongue Image Analyzing System (TIAS)) for performing tongue diagnosis. The key characteristic of the tongue imaging method in TIAS is the exclusion of the influence of external light using an integrating sphere to achieve an evenly distributed light intensity with a halogen light source (Moritex Inc., MHAB-150W, color temperature 3200 K). Further, TIAS can remove the gloss of the tongue surface from its images. Our prior study investigated the use of spectral camera imaging [10]. We confirmed the relationships between the Kampo concept of* Oketsu *and both liver function and thyroid function in blood samples as measured by wavelength values [11–13]. Subsequently, in aiming to further promote TIAS, we changed from a spectral camera to a digital camera (Lumenera Inc., Lw115C, 1280 × 1024 pixels, Color CMOS sensor), although we are still using the basic data from the spectral camera. We changed because digital cameras are cheap and the color is superior for viewing purposes. For the quantitative measurement methods in TIAS, RGB values of digital camera images were converted into CIE1976 *L*∗*a*∗*b*∗ color space values. Imaging by TIAS was confirmed to be stable for 3 weeks [14]. As mentioned above, our method has made it possible to perform stable quantitative measurement of tongue images by TIAS, and we have almost solved the problem of EF.

As far as we know, there are no other reports about subjective factors (SF) in TCD; the problem of SF has remained unclear. In tongue color diagnosis, age, gender, difference in color discrimination, and experience and knowledge in Kampo medicine are thought to be important influences. Thus, we set out to examine the influence of these factors. We studied the relation of age, gender, color discrimination, and duration of Kampo experience on TCD. One method to evaluate color discrimination is the Farnsworth-Munsell 100 Hue test (Hue test). The Hue test has been used for many years in industrial fields to check color discrimination. In various other fields, many studies on color discrimination have been reported using the Hue test. [15, 16]. The Hue test was evaluated for color discrimination of patients with optic neuritis in ophthalmology [17, 18]. And, the Hue test has been used by neuroscientists to study color discrimination and occipital lobe function in patients with Parkinson's disease and pituitary adenoma [19–21]. The Hue test was first devised by Farnsworth in 1943, and the present 85 colored-caps version was improved in 1957 [22]. The color caps are divided into four hues, and the 85 caps are arranged into four boxes, each containing a fixed anchor cap at both ends of each box. One box consists of 22 caps, and the other three boxes consist of 21 caps each. Color discrimination is evaluated when the subject attempts to arrange the caps into the correct hue order. The total Hue score is calculated by the number of misplacements. Thus, a lower Hue score indicates better color discrimination.

The purpose of this study was to reveal the SF involved in Kampo tongue diagnosis. We recorded data about age, gender, duration of Kampo experience, and primary occupations in Kampo medical practitioners. We evaluated color discrimination by the Hue test. Simultaneously, we examined the individual discrepancies in TCD using tongue images in which the color was adjusted by computer processing, and we studied the relationships of age, gender, color discrimination, and duration of Kampo experience with these results.

## 2. Subjects and Methods

### 2.1. Subjects

**Figure 1:**
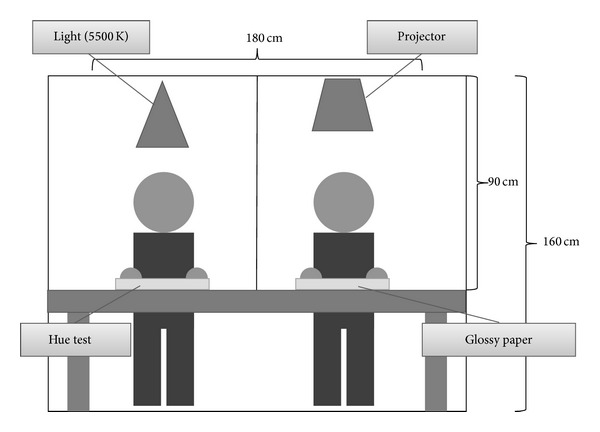
The experimental environment in this study. The experiments were conducted in a dark room with a single light source. For the Hue test, the light was positioned above so that the angle of illumination would be 90° and the angle of Hue test viewing would be approximately 60°. For the tongue color diagnosis, full-color tongue images were projected onto glossy paper by a projector.

The subjects were 68 Kampo medical practitioners (48 males, 20 females). First, we questioned the subjects about their age, gender, duration of Kampo experience, and primary occupation. In order to maintain advanced color reproducibility, we had to exclude the influence of external light in the experimental environment. Thus, the experiments were conducted in a dark room (Figure 1). All subjects continuously performed the Hue test and TCD using tongue images.

### 2.2. Hue Test

**Figure 2:**
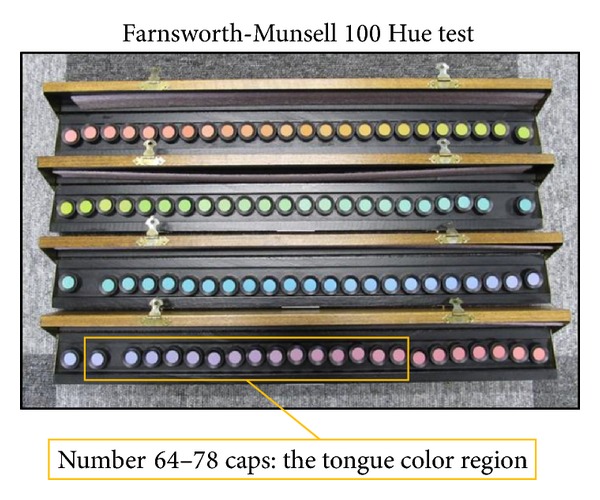
Color discrimination was evaluated by the Farnsworth-Munsell 100 Hue Test. The color discrimination is evaluated based on the subject's attempt to rearrange the caps into the correct hue order. Total Hue scores are calculated as the number of misplacements, and a lower score therefore indicates better color discrimination. The tongue color regions (TCRs) correspond to caps number 64–78.

We used artificial solar illumination (SERIC Inc., XC-19, 5500K) for the Farnsworth-Munsell 100 Hue test (SAKATA Inc., Farnsworth-Munsell 100 Hue test Munsell color) (Figure 2). The illumination was set on the ceiling of the darkroom, so that the angle of illumination could be about 90° and the angle of viewing could be about 60°. The subjects were ordered to rearrange the color caps of one color phase placed randomly in one slim-line box in correct order in two minutes. They performed this task on all four color phases; that is, they completed the Hue test. We calculated the Hue score according to the number of caps rearranged incorrectly compared with the correct orders of color phases. There were three levels of color discrimination ability: the superior-ability group (Hue score 0–16), normal-ability group (Hue score 20–100), and low-ability group (Hue score more than 100) [22]. In addition, we measured actual values of all the color caps in CIE 1976* L*∗*a*∗*b*∗ color space using the spectroradiometer (KONICA MINOLTA Inc., CS-1000A) in the experimental environment. The result shows that the tongue colors used in this experiment corresponded to those of color caps number 64–78 in CIE 1976* L*∗*a*∗*b*∗ color space (Figure 2). Therefore, we established a tongue color region (TCR) as the number 64–78 color caps region, and we also examined the relation between each influence factor and TCR.

### 2.3. Tongue Color Diagnosis (TCD) of Tongue Images

**Figure 3:**
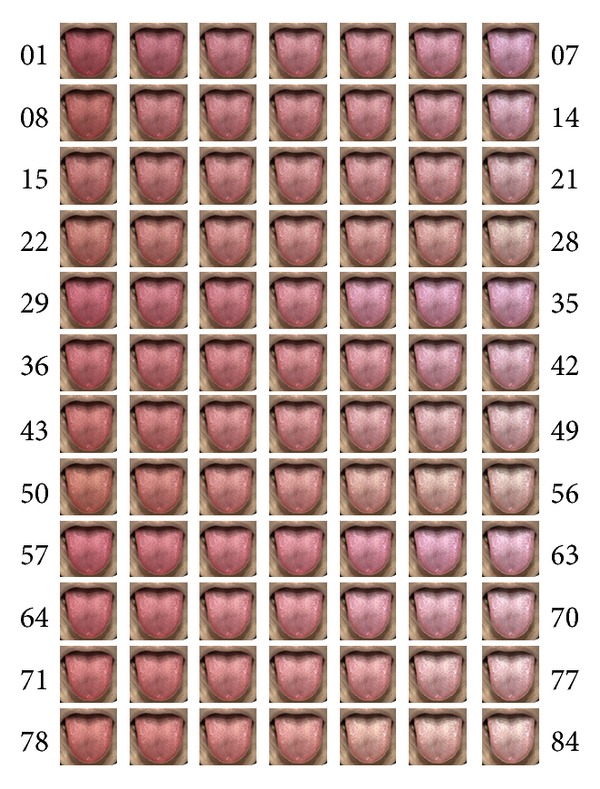
Tongue images 01–84.

**Figure 4:**
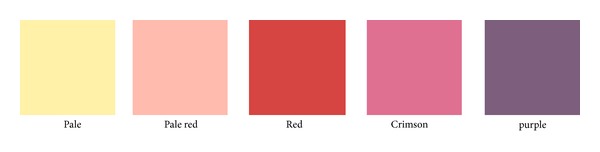
Tongue color diagnosis included five colors: pale, pale red, red, crimson, and purple.

In creating the tongue color images, we used 1551 tongue images taken by TIAS. In order to determine the distribution of the colors of the 1551 tongue images, we performed principal component analysis. By determining the color using principal component axes, it was possible to set the color gamut of the tongue without deviating far from the tongue color. The distance of the color becomes a constant interval by dividing the tongue color gamut into a 7 : 4 : 3 ratios and the 84 (7 × 4 × 3) tongue images were obtained from it (images 01–84: Figure 3). Furthermore, we measured actual values of the tongue colors on the tongue image color chart in CIE 1976* L*∗*a*∗*b*∗ color space in an experimental environment. The tongue color images were projected onto glossy paper by a projector (EPSON Inc., EB-1761W), and subjects were asked to diagnose the tongue color in each of the 84 tongue images. The color of the TCD was selected from among five designations: pale, pale red, red, crimson, and purple (Figure 4) [23].

### 2.4. The Ethics and Statistical Analysis

We obtained informed consent for this experiment from all subjects using descriptive text.

The data were analyzed by Student's* t*-test when they were assumed to be homoscedastic by the* F*-test. If the data could not be assumed to be homoscedastic by the* F*-test, they were analyzed by Welch's test. When the data were not consecutive variables, they were analyzed by the *χ* ^2^-test. In each analysis, the significance level was set at less than 5%. We used the Pearson product-moment correlation coefficient.

## 3. Results

### 3.1. Subjects

In this study, we obtained data from 68 Kampo medical practitioners. Their ages ranged from 27 to 69. The average age was 44.3 ± 9.1 among all subjects, 45.9 ± 8.9 among males and 40.5 ± 8.4 among females; the median age was 43. The subjects' duration of Kampo medical experience averaged 12.1 ± 9.5 years, ranged from 1 to 40, and had a median value of 10. The subjects consisted of 52 medical doctors, 6 acupuncturists, and 10 pharmacists who made tongue diagnosis in daily operations (Table 1). There was a positive correlation between the ages and the duration of Kampo medical experience (*r* = 0.753). There were fewer females than males in the age group ≥43 years and in the group with ≥10 years of Kampo medical experience.

### 3.2. Hue Test Color Discrimination in the Entire Region and the Number 64–78 Region

The entire region of Hue scores (EHS) ranged from 4 to 138, with an average of 39.2 ± 25.4 and median value of 30. There were 12 subjects with superior ability, 54 subjects with normal ability, and 2 subjects with low ability of color discrimination according to the Hue score. The Hue scores for the number 64–78 caps ranged from 0 to 36, with an average of 4.4 ± 6.6, and a median value of 2.

**Figure 5:**
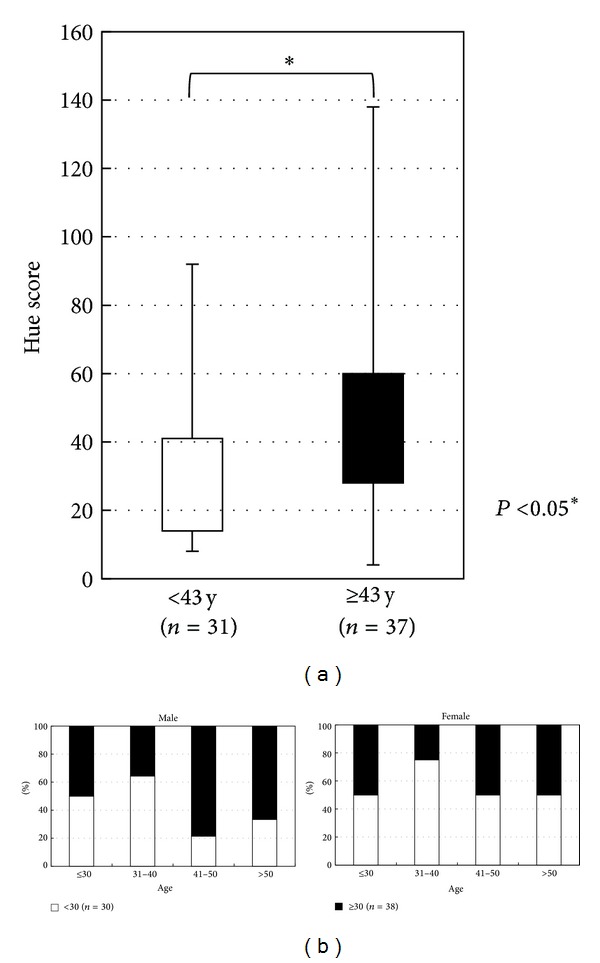
(a) The <43 years group had significantly lower average Hue scores than the ≥43 years age group (*t*-test, *P* = 0.012). (b) The rate of subjects with Hue scores of <30 and ≥30 was compared between genders and separated by age. There were no significant differences between males and females.

**Figure 6:**
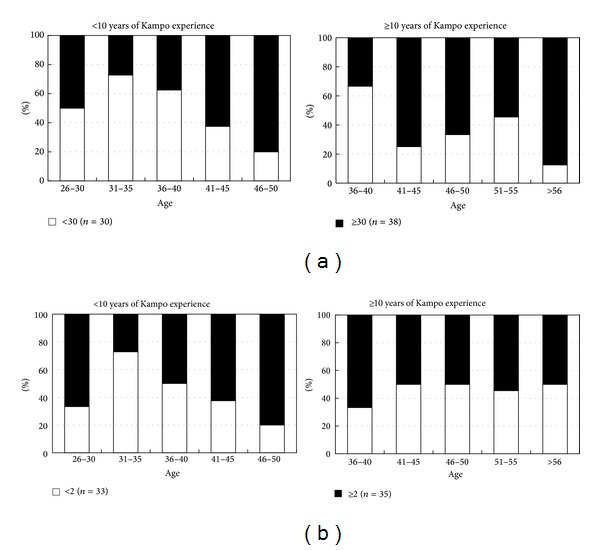
(a) The <10 years of Kampo experience group and ≥10 years of Kampo experience group were divided into EHS < 30 (*n* = 30) and EHS ≥ 30 (*n* = 38) in each age group. There was no significant relationship between Kampo experience and high/low EHS. (b) The <10 years of Kampo experience group and ≥10 years of Kampo experience group were divided into 64–78 HS < 2 (*n* = 33) and 64–78 HS ≥ 2 (*n* = 35) for each age group. A significant difference was observed in the 64–78 HS < 2 group between the <10 years and ≥10 years of experience groups (*χ* ^2^-test, *P* < 0.01). The ages were higher in the ≥10 years of Kampo experience group, but the ratio of 64–78 HS < 2 did not decrease.

EHSs were analyzed in terms of age, gender, and duration of Kampo medical experience. The group of <43 years old (*n* = 31) had a significantly lower EHS average (better color discrimination) than those ≥43 years old (*n* = 37) (*t*-test, *P* = 0.012) (Figure 5(a)). With regard to gender, there was no significant difference between the number of men and women in the groups with scores <30 and ≥30, stratified by age (Figure 5(b)). There was no significant difference between the rate of inexperienced (<10 years) and experienced (≥10 years) Kampo practitioners in the groups with scores of <30 (*n* = 30) and ≥30 (*n* = 38) stratified by age (Figure 6(a)).

The number 64–78 region of Hue scores (64–78 HS) was considered to correspond to the TCR. In the same way as described for EHSs above, 64–78 HS were analyzed with regard to age, gender, and duration of Kampo experience. No significant difference in the 64–78 HS between the age groups <43 years (*n* = 31) and ≥43 years (*n* = 37) was found (*t*-test, *P* = 0.257). Analyzing the mean 64–78 HS for each gender in each age group, no significant difference was found between males and females. In terms of Kampo experience, we compared the ratios of experienced (≥10 years) and inexperienced (<10 years) Kampo practitioners in the group with 64–78 HS < 2 (*n* = 33) and that with 64–78 HS ≥ 2 (*n* = 35) (Figure 6(b)). A significant difference was found in the ratios in each score category between the two groups (*χ* ^2^-test, *P* < 0.01). In workers with <10 years of Kampo experience, age had a deleterious effect on color discrimination, with those >30 years old having a smaller ratio of good 64–78 HS scores (64–78 HS < 2). However, in the group with ≥10 years of Kampo experience, the ratio of 64–78 HS < 2 did not decrease. This tongue-color-specific region was the only one for which significant differences in color discrimination were found between workers with Kampo experience for <10 years and those with Kampo experience for ≥10 years; furthermore, in other color regions, Hue scores uniformly increased with aging.

### 3.3. Tongue Color Diagnosis of Tongue Images

**Figure 7:**
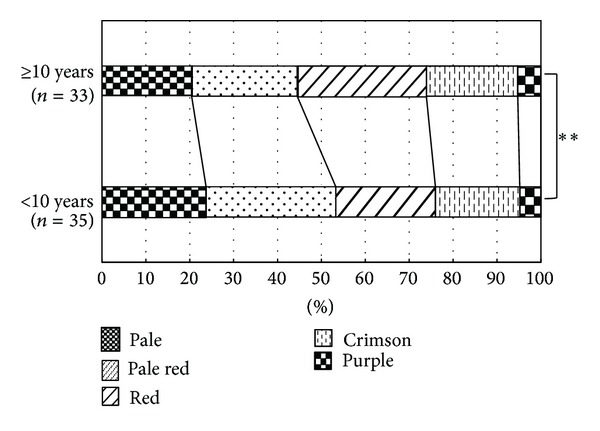
Comparison of TCD and duration of Kampo experience. The distribution of TCD showed a significant difference in the comparison of the <10 years and ≥10 years of Kampo experience groups. All *P* values were obtained by *χ*²-test. *P < 0.05,**P < 0.01.

**Figure 8:**
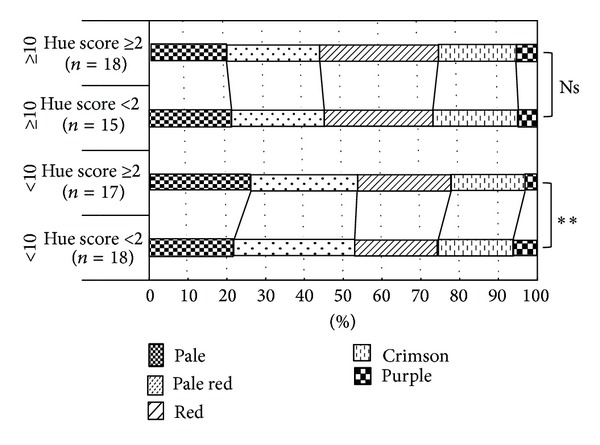
Comparison of TCD with 64–78 HS and duration of Kampo experience. The distribution of TCD was significantly different in the 64–78 HS < 2 and ≥2 groups within the <10 years of Kampo experience group. On the other hand, no significant difference was found in the ≥10 years of Kampo experience group. All *P* values were obtained by *χ*²-test. *P < 0.05, **P < 0.01, Ns: no significance.

We examined the total number of answers of each tongue color for TCD of the tongue images (images 1–84) projected onto glossy paper. The total number of answers was 5712 (84 images × 68 subjects). The cumulative numbers of answers of each tongue color were as follows: pale 1265, pale red 1536, red 1482, crimson 1142, and purple 287. For each tongue color we compared the answer distributions with regard to age, gender, color discrimination (EHS and 64–78 HS scores), and duration of Kampo experience. There were no significant differences between age, gender, and color discrimination abilities (EHS and 64–78 HS scores) for TCD of the tongue images. However, the distribution of TCD was significantly different between workers with <10 years of Kampo experience (*n* = 35) and those with ≥10 years of experience (*n* = 33) (*χ* ^2^-test, *P* < 0.01) (Figure 7). Incidentally, there was no significant difference between other durations of Kampo experience. Further, we examined the relationship with TCD of the 64–78 HS groups and the duration of Kampo experience. TCDs were compared for the groups with 64–78 HS < 2 (*n* = 18) and 64–78 HS ≥ 2 (*n* = 17), first in the group with <10 years of Kampo experience, and then in the group with ≥10 years of Kampo experience (Figure 8). As a result, the distribution of TCD was significantly different between 64–78 HS < 2 and ≥2 in workers with <10 years of Kampo experience (*χ* ^2^-test, *P* < 0.01), but not for with ≥ 10 years of Kampo experience.

## 4. Discussion

We studied the relationships between color discrimination on the one hand and age, gender, and duration of Kampo experience on the other. We found that overall color discrimination was associated with age but that the color discrimination of the tongue color region (TCR) was associated with duration of Kampo experience. Further, we found that duration of Kampo experience influenced TCD.

TCD and color discrimination measurement require high color reproducibility. The reason is that color is determined by both the illumination light source and the characteristics of the object. Hence, the settings of the illumination light source, the light source position, and the observation viewpoint are important in order to obtain an accurate representation of the color. Zahiruddin et al. compared two conditions for the Hue test, the conventional observation method and observation under ambient room light. They recognized that Hue scores differed in the two conditions [24]. In order to control the conditions, we followed the method of D. Farnsworth, in which the angle of illumination was vertical at 90° and the angle of Hue test viewing was about 60° in an otherwise dark room [22]. On the other hand, the human visual system has two modes of appearance of the color; that is, the two visual characteristics are the light source color mode and the object color mode [25]. Usually, we observe tongue color in the object color mode. Therefore, in this experiment, we observed the tongue color in the object color mode using a projector. The measurement value in CIE 1976* L*∗*a*∗*b*∗ color space had been preset using the color caps of Hue test and the tongue color images. However, as the color is affected by the experimental environment, we also measured actual values (CIE 1976* L*∗*a*∗*b*∗) in the experimental environment. We set a TCR that matched the color caps of the Hue test and the tongue color images in the actual measurement value. Number 64–78 caps from the Hue test were considered equivalent to the color of tongue diagnosis. The method used to identify the red-green or blue-yellow area in the Hue test has been described in previous reports [17, 26]. In this study, different results were obtained when the data of this restricted area of color were evaluated instead of the entire area of the Hue test.

In general, it has been reported that discrimination of colors represented by the entire Hue test region worsens with aging [27–29]. Similar results were observed in this study. In Kinnear and Sahraie, the average Hue scores decreased gradually to about 20 years of age, but after 20 the scores increased with aging (color discrimination worsened). The average Hue score was found to increase greatly at 50 years or more [27]. Roy et al. reported the same results [28, 29]. However, the average Hue scores in our study are lower than those in these previous reports. Likewise, the rate of increase of average Hue score for subject's ≥50 years in this study was lower than in these reports. We found an effect in this study whereby the color discrimination of the TCR does not suffer an age-related worsening in Kampo practitioners with ≥10 years of experience. We think this effect explains our results.

There was no significant difference in color discrimination by gender (Hue test entire region and TCR). Some reports have compared color discrimination by gender using the Hue test [26, 30]. Rigby et al. examined Hue test color discrimination by gender in pathologists [30] and found no significant difference between the scores of 23 males and 7 females in the 20–45 years age range. Moreover, Koçtekin et al. considered specific regions of Hue test in the dominant eye and the opposite side of eye of medical students [26]. The subjects were 31 males and 19 females whose mean age was 21 ± 2 years of age. Again, there was no significant difference between males and females in their study. Although the designs and purposes of these reports were different from those in this study, the results are consistent. Therefore, although the male-to-female ratio of this study was not 1 : 1, we think the effect of this bias is small.

In the TCD, no association was found between age, gender, and color discrimination (Hue test entire region and TCR). However, a significant difference in TCD was recognized between inexperienced (<10 years) and experienced (≥10 years) practitioners. Thus, an association was suggested between TCD and duration of Kampo experience. The inexperienced group tended to evaluate the pale-red area more broadly than the experienced group. Conversely, the experienced group tended to evaluate the pale and the pale-red areas more narrowly and to evaluate the red areas more broadly. Thus, there is a possibility that the ability to identify the pale-red area (normal tongue color area) is increased by gaining experience in Kampo medicine. We examined the TCD results by two factors, color discrimination of TCR and duration of Kampo experience. In the inexperienced practitioner group (<10 years), TCD results differed depending on the color discrimination of TCR. On the other hand, in the experienced group (≥10 years), TCD results were not affected by the color discrimination of TCR, but rather the TCD results became constant. Therefore, until having enough TCD, training of Kampo medicine may be needed for 10 years or more.

Actual clinical doctors diagnose tongue color independently. For this reason, we think this study is valuable because it involved the participation of many Kampo medical practitioners. Moreover, the finding that the TCD is affected by duration of Kampo experience is novel. Using this new finding, it may be possible to obtain more accurate results in the selection of tongue color diagnosis. We need to consider the duration of Kampo experience when judging tongue color findings. Further, in Kampo medicine education, age or color discrimination ability should not be considered a barrier, as experience and training can make up for these deficits. This study suggests the importance of TCD study, which we hope will progress in future. Finally, we believe this study can contribute to the standardization of tongue diagnosis in Kampo medicine.

## 5. Conclusions

Overall color discrimination worsened with aging, but the ability of tongue color diagnosis was not affected by aging or color discrimination ability. The ability of tongue color diagnosis and indeed ability to discern colors in the tongue color region do not degrade in those with Kampo experience. These results suggest the importance of studying tongue color diagnosis, and they are expected to contribute to the standardization of tongue diagnosis and Kampo medical education in the future.

## Figures and Tables

**Table 1:** Comparison of duration of Kampo experience with subjects' age, gender, and occupation.

	Duration of Kampo experience		*P* value
	<10 years	≧10 years	
Age			
<43 years	26	5	0.000**
≥43 years	9	28	0.001**
Gender			
Male	19	29	0.099
Female	16	4	0.011*
Occupation			
M.D.	22	30	0.186
Not M.D.	13	3	0.017*

M.D.: medical doctor.

All *P* values were obtained by *χ*²-test. *P < 0.05, **P < 0.01.
